# Cross-sectional variations of white and grey matter in older hypertensive patients with subjective memory complaints

**DOI:** 10.1016/j.nicl.2017.12.024

**Published:** 2017-12-18

**Authors:** Ahmed Chetouani, Mohammad B. Chawki, Gabriela Hossu, Anna Kearney-Schwartz, Florence Chauveau-Beuret, Serge Bracard, Veronique Roch, Vincent Lebon, Pierre-Yves Marie, Athanase Benetos, Laure Joly, Antoine Verger

**Affiliations:** a CHU-Nancy, Department of Nuclear Medicine & Nancyclotep Imaging platform, Nancy, F-54000, France; b INSERM U947, Nancy F-54000, France; c INSERM, Centre d'Investigation Clinique CIC-IT, 1433, Nancy F-54000, France; d CHU-Nancy, Department of Geriatric Medicine, Nancy, F-54000, France; e CHU-Nancy, Department of Neuroradiology, Nancy, F-54000, France; f INSERM, Centre d'Investigation Clinique CIC-P 9501, Nancy, F-54000, France; g Department of Nuclear Medicine Frédéric Joliot, CEA, Orsay, F-91400, France; h INSERM, U1116, Nancy, F-54000, France

**Keywords:** Diffusion tensor imaging, FDG PET, Elderly, Subjective memory complaint, HTA

## Abstract

Mild cognitive impairment and Alzheimer's dementia involve a grey matter disease, quantifiable by ^18^F-Fluorodeoxyglucose positron emission tomography (FDG-PET), but also white matter damage, evidenced by diffusion tensor magnetic resonance imaging (DTI), which may play an additional pathogenic role. This study aimed to determine whether such DTI and PET variations are also interrelated in a high-risk population of older hypertensive patients with only subjective memory complaints (SMC).

Sixty older hypertensive patients (75 ± 5 years) with SMC were referred to DTI and FDG-PET brain imaging, executive and memory tests, as well as peripheral and central blood pressure (BP) measurements. Mean apparent diffusion coefficient (ADC_mean_) was determined in overall white matter and correlated with the grey matter distribution of the metabolic rate of glucose (CMRGlc) using whole-brain voxel-based analyses of FDG-PET images.

ADC_mean_ was variable between individuals, ranging from 0.82 to 1.01.10^− 3^ mm^2^ sec^− 1^, and mainly in relation with CMRGlc of areas involved in Alzheimer's disease such as internal temporal areas, posterior associative junctions, posterior cingulum but also insulo-opercular areas (global correlation coefficient: − 0.577, *p* < 0.001). Both the ADC_mean_ and CMRGlc of the interrelated grey matter areas were additionally and concordantly linked to the results of executive and memory tests and to systolic central BP (all *p* < 0.05).

Altogether, our findings show that cross-sectional variations in overall white brain matter are linked to the metabolism of Alzheimer-like cortical areas and to cognitive performance in older hypertensive patients with only subjective memory complaints. Additional relationships with central BP strengthen the hypothesis of a contributing pathogenic role of hypertension.

## Highlights

• Changes in grey and white matter are interrelated in elderly hypertensive patients.
• They can be documented in the absence of any objective memory complaint.
• These interrelationships are associated with cognitive test results.
• The interrelated grey matter areas are likely to define an Alzheimer-like pattern.
• These interrelationships are linked to the level of central blood pressure.

## 1 Introduction

Subjective cognitive impairment (SCI) is common in the elderly, and may serve as a symptomatic indicator of a precursor stage of Alzheimer's dementia (AD), even if subtle cognitive decline is difficult to detect on standardized cognitive testing (Jessen et al., 2014). While this condition is not considered to be a definite neurodegenerative process such as mild cognitive impairment (MCI) or AD, it may precede a further cognitive decline and the development of dementia (Kielb et al., 2017).

In addition, impaired cognitive performance has been associated with cardiovascular (CV) risk factors such as hypertension (Ferreira et al., 2017, Muller et al., 2007, Rafnsson et al., 2007), in keeping with our recent observation that brain remodeling with age is linked to the level of central pulse pressure (Verger et al., 2015). Thus, older hypertensive patients with SCI may constitute a particularly high-risk group for subsequent dementia and may therefore benefit from dedicated modalities of medical management and of early diagnosis.

Recent advances in MRI and PET imaging modalities can detect early changes in brain structure and/or metabolism, before the stage of dementia. Among these, diffusion tensor imaging (DTI), through the mean apparent diffusion coefficient (ADC_mean_), may be particularly useful for the early diagnosis of neurodegenerative disorders (Sali et al., 2013). This DTI-derived parameter provides a measurement of diffusion rate and its global value is more closely linked to the neurodegenerative process than local values (Kin et al., 2006). White matter ADC_mean_ is indeed commonly increased in neurodegenerative diseases owing to the loss of axonal myelin and the disruption of cell membranes (Kin et al., 2006).

^18^F-Fluorodeoxyglucose positron emission tomography (^18^F-FDG PET) is also a useful imaging method in this setting, owing to its ability to quantify neuronal activity through the glycolytic metabolism of the brain grey matter (Magistretti and Allaman, 2015). ^18^F-FDG PET is moreover increasingly used for the early diagnosis of dementia and predementia states and more precisely, for detecting the degenerative component of these diseases, in accordance with the recommendations of the international Alzheimer's Association (McKhann et al., 2011). ^18^F-FDG PET abnormalities, which are documented in AD or MCI patients, were recently shown to correlate with the microstructural white matter changes observed with DTI (Yakushev et al., 2011a, Zimny et al., 2015, Walhovd et al., 2009). To date, however, it is not known whether these interrelated white and grey matter changes are also present in patients with only subjective memory complaints, before the stage of any objective cognitive impairment.

In light of the above, this dual DTI and ^18^F-FDG PET study aimed to determine whether cross-sectional variations within the white and grey brain matters are also associated before the stage of any objective cognitive impairment, in a high-risk population of older hypertensive patients with only subjective memory complaints.

## 2 Materials and methods

### 2.1 Subjects

This ancillary PET/MRI study was extracted from the ADELAHYDE longitudinal single-center study, which aimed at identifying factors associated with cognitive decline and white matter diseases in older hypertensive patients with subjective memory complaints (Analyse des DEterminants génétiques et environnementaux de la Leucoaraiose dans une population de sujets Agés Hypertendus présentant des troubles cognitifs DEbutants; ClinicalTrials.gov Identifier: NCT01351961). Inclusion and exclusion criteria have already been detailed elsewhere (Kearney-Schwartz et al., 2009).

A total of 131 patients participated to the ADELAHYDE-2 study and results of the present study were extracted from the second control visit which comprised a medical examination with measurement of central blood pressure (BP), various neuropsychological tests and a brain MRI (Ferreira et al., 2017). Among these 131 patients, 71 accepted to undergo an additional investigation by brain ^18^F-FDG-PET (local Ethics Committee (CPP) agreement no 2010-A01399-30), although the study population was finally restricted to 60 patients, 11 being excluded for technical issues at MRI (3 without any DTI acquisition and 2 with incorrect acquisitions) or for a significant cognitive impairment and a high probability of MCI (*n* = 4) or AD (*n* = 2) based on neuropsychological tests (score < 25 for Mini-Mental State Examination or < 17 for free recall or < 40 for total recall of the Grober-Buschke tests (Sarazin et al., 2007)).

All investigations were planned on the same day, except for the brain MRI, which was performed in the following 3 months.

### 2.2 Clinical cardiovascular and neuropsychological assessments

Peripheral brachial BP was measured in the supine position with an oscillometric semiautomatic device (Omron 705IT, Kyoto, Japan) after a minimum 10-min rest period. Systolic, diastolic, pulse and mean BPs were recorded three to four times and averaged for subsequent analyses.

Central BP was determined in 56 patients (24 women) by the transcutaneous analysis of the carotid pulse wave with an applanation tonometer (Joly et al., 2009, Salvi et al., 2004). Carotid pressures were deemed as a close surrogate of central pressures and calibrated with the diastolic and mean brachial BP values (the differences in diastolic and mean arterial pressure are minimal throughout the arterial tree) (Verger et al., 2015).

Neuropsychological assessment was comprised of: 1) a Mini-Mental State Examination test for global cognition (Folstein et al., 1975), 2) the Free and Cued recall tests (i.e. (Hofman et al., 1997) a French equivalent of the Grober-Buschke test for the capacities of encoding and consolidation, as well as for the efficiency of the recovery mechanisms (Grober et al., 1988), 3) a Benton Visual Retention Test for visuospatial capacities (Spreen and Benton, 1963), 4) the Verbal Fluency Test for executive function and long-term verbal memory (Strauss et al., 2006), and 5) the Trail Making Tests for visual attention and task switching (Ashendorf et al., 2008, Greenlief et al., 1985).

### 2.3 MRI acquisition and analysis

All data were acquired on a 1.5-T magnet (Signa HDxt, GE Healthcare, Milwaukee, WI, USA) with an 8-element receive head coil (Invivo Corp, Orlando, FL). The protocol involved the recording of a 3-dimensional T1-weighted sequence with the following parameters: slice thickness 1.4 mm; TE/TR/TI = 5/12/350 ms, field of view 240 mm (matrix size 256 × 256), and Fluid-Attenuated Inversion Recovery (FLAIR) images, with the following parameters: slice thickness 5 mm, TE/TR/TI = 158/10000/2300 ms, field of view 240 mm (matrix size 288 × 224). White matter hyperintensities of presumed vascular origin were assessed on the FLAIR images by a blinded experimented radiologist (SB) using the Fazekas score, corresponding to the sum of periventricular and deep white matter hyperintensity ratings (Fazekas et al., 1987).

A DTI axial acquisition was also performed with the following parameters: 15 non-collinear gradient directions with b = 1000 s/mm^2^, one b = 0 reference image, contiguous slices of 5 mm thickness; TE/TR = 72–100/9.600 ms, field of view 240 mm (matrix size of 128 × 128) covering the entire brain and cerebellum.

An automated parcellation of the subcortical white matter was obtained on the 3D T1-weighted images from each patient and by using the “-recon-all” processing pipeline of the FreeSurfer software version 5.2. This parcellation was thereafter applied to the DTI images through a co-registration and a transformation in the 3D-T1 space of the DTI images (“Dt_recon” function of the FreeSurfer software). Finally, ADC_mean_ values were obtained from the total white matter and from the different lobes using a subcortical white matter parcellation atlas “wmparc” (Salat et al., 2009).

### 2.4 ^18^F-FDG-PET recording and analysis

The ^18^F-FDG-PET images were recorded on a Biograph™ 6 hybrid PET/Computed Tomography (CT) system (Siemens Medical Solutions, Erlangen, Germany). Patients were fasted for at least 6 h prior to the injection of 4 to 5 MBq/kg of ^18^F-FDG and subsequently placed in a quiet environment with eyes closed. Fifty minutes later, a 3-dimensional Computed Tomography (CT) of the brain was recorded and immediately followed by a 3D PET brain recording over a 15 min period. Images were reconstructed with an iterative 3-dimensional Ordered Subset Expected Minimization (OSEM) method, corrected for attenuation and diffusion, and displayed through 2.7 × 2.7 × 2.7 mm^3^ voxels.

A whole-brain statistical analysis was performed at the voxel level using the SPM8 software (Wellcome Department of Cognitive Neurology, University College, London, UK). ^18^F-FDG PET images were spatially normalized onto an adaptive template derived from the MR and ^18^F-FDG PET images of our subjects, as previously reported (25). After normalization to the adaptive template, ^18^F-FDG PET images were smoothed with a Gaussian filter (FWHM 8 × 8 × 8 mm^3^) and normalized to through intensity ratios relative to mean cerebellar activity (Van Der Gucht et al., 2015). Thereafter, the PET images were corrected for partial volume effect using the grey matter volume segmented from co-registered MR images for each patient (Gutierrez et al., 2012).

The SPM linear regression models, used for correlating ^18^F-FDG metabolism from the brain grey matter voxels with the ADC_mean_ values from overall white matter as well as for each lobe, were obtained: 1) at a threshold (voxel-level significance) of *p* < 0.005, 2) with a correction for cluster volume and using the expected voxels per cluster provided by SPM, in order to avoid type II errors as recommended (Lieberman and Cunningham, 2009) and 3) by using age and gender as covariates. The anatomical localizations of significant clusters were identified using the MNI (Montreal National Institute) atlas.

Finally, mean relative values of the cerebral metabolic rate of glucose (CMRGlc) of the combination of clusters interrelated with ADC_mean_ of overall white matter were extracted for each patient.

### 2.5 Statistical analysis

Quantitative variables are expressed as means ± standard deviations, and categorical variables as percentages. Student-*t*-tests were performed for the unpaired 2-group comparison of quantitative variables. Pearson coefficients were used to assess the correlation between ADC_mean_, CMRGlc, cardiovascular parameters and/or neuropsychological test scores. A *p* < 0.05 was determined as significant. Statistical analyses were performed with SPSS® 20.0 software. The statistical analyses performed with the SPM software have been already detailed above.

## 3 Results

### 3.1 Population characteristics and main recorded data

The study population involved 60 patients (76 ± 5.1 years old, 34 women) and all were treated with at least one hypertensive medication, in accordance with the inclusion criterion. Seventeen (28%) had an uncontrolled hypertension, as defined for older subjects by a brachial systolic BP higher than 150 mm Hg (Mancia et al., 2014). On FLAIR-MRI images, the severity of white matter hyperintensities of presumed vascular origin was absent to mild (Fazekas scores 0 to 2) in 26 patients (43%), moderate (scores 3 to 4) in 18 (30%) and severe (scores 5 to 6) in 16 (27%). The main recorded quantitative data are summarized in Table 1 for the overall population along with a comparison between men and women. No difference was documented between men and women except for certain neuropsychological tests which were less well performed by men (Gröber and Buschke, *p* < 0.01 and Trail Making Tests B and B-A, *p* < 0.04).

**Table 1:** Main recorded quantitative data with comparison between men and women.

	Overall (*n* = 60)	Women (*n* = 34)	Men (*n* = 26)	*p*-Value
Clinical and BP variables				
Age (years)	76 ± 5.1	75.4 ± 4.6	76.5 ± 5.6	0.40
Duration of hypertension (years)	21.8 ± 8	22.6 ± 8.6	20.7 ± 7.3	0.46
Number of antihypertensive medications	2.0 ± 0.9	2.0 ± 0.9	1.9 ± 0.9	0.78
Peripheral systolic BP (mm Hg)	142.6 ± 17.3	142.7 ± 16.9	142.5 ± 18.2	0.20
Peripheral diastolic BP (mm Hg)	73.3 ± 9.6	72.6 ± 8.9	74.2 ± 10.6	0.15
Peripheral pulse pressure (mm Hg)	69.3 ± 12.7	70.1 ± 14.1	68.3 ± 10.6	0.58
Central systolic BP (mm Hg)†	134.1 ± 18.9	132.5 ± 19.2	136.3 ± 18.7	0.36
Central pulse pressure (mm Hg)†	59.9 ± 16.1	59.2 ± 17.5	61 ± 14.1	0.87
Results of neuropsychological tests				
MMSE (/30)	29.4 ± 1.1	29.6 ± 0.8	29.2 ± 1.4	0.25
Benton score	12.8 ± 6.8	14 ± 6.9	11.2 ± 6.5	0.12
Gröber and Buschke free recall (/48)	28.4 ± 7.5	31 ± 7	25 ± 6.9⁎	< 0.01
Gröber and Buschke cued recall	17.6 ± 5.9	15.5 ± 5.8	20.3 ± 5⁎	< 0.01
Trail making test A (sec)	47.9 ± 14.8	46.4 ± 12.3	49.8 ± 17.4	0.40
Trail making test B (sec)	101.1 ± 48.2	88.4 ± 33.1	116.8 ± 59⁎	0.04
Trail making test B-A (sec)	53.2 ± 38.6	42 ± 27.2	67 ± 46.1⁎	0.02
Verbal fluency test score (P letter)	12 ± 4.2	12.6 ± 4.7	11.3 ± 3.5	0.23
Verbal fluency test score (R letter)	11.3 ± 4.3	11.8 ± 4.4	10.8 ± 4.1	0.38
ADC_mean_ from overall white matter (10^− 3^ mm^2^ sec^− 1^)	0.89 ± 0.05	0.89 ± 0.04	0.89 ± 0.05	0.45
Frontal ADC_mean_	0.89 ± 0.05	0.89 ± 0.05	0.90 ± 0.05	0.19
Parietal ADC_mean_	0.85 ± 0.04	0.86 ± 0.05	0.85 ± 0.04	0.61
Temporal ADC_mean_	0.85 ± 0.04	0.85 ± 0.04	0.85 ± 0.04	0.45
Occipital ADC_mean_	0.85 ± 0.04	0.85 ± 0.03	0.84 ± 0.04	0.17
Fazekas score	3.3 ± 1.6	3.4 ± 1.5	3.2 ± 1.7	0.66

BP, blood pressure; MMSE, mini-mental-state-examination; ADC_mean_, mean apparent diffusion coefficient.

⁎ p < 0.05 for comparison with women.

† Data available for only 56 patients (24 women).

### 3.2 Relationships between the inter-individual variations in ADC_mean_ and in CMRGlc

**Fig. 1:**
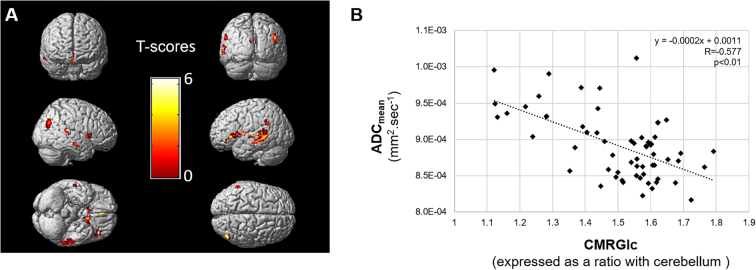
A Three-dimensional volume rendering images representing the grey matter areas for which the metabolic rate of glucose (CMRGlc) was significantly (*p* < 0.005, with minimum cluster size of 39 voxels) and negatively correlated with the mean apparent diffusion coefficient (ADC_mean_) of the overall white matter and by using age and gender as covariates (A). B: Linear and inverse relationship between the mean apparent diffusion coefficient (ADC_mean_) of the overall white matter and the global metabolic rate of glucose (CMRGlc) of the grey matter voxels involved in the significant area displayed in Fig. 1A.

The ADC_mean_ of the overall white matter was variable between patients, ranging from 0.82 to 1.01. 10^− 3^ mm^2^ sec^− 1^, and as detailed in Fig. 1A and Table 2, this ADC_mean_ was strongly and inversely related to the CMRGlc of areas extending over 23.3 cm^3^ and involving internal temporal areas (BA 36), posterior associative junctions (BA 18-19-21-22-39-40), posterior cingulum (BA 30-31) and insulo-opercular areas (BA 11-13-47), independently of the additional influences of age and gender. The strength of the link between the ADC_mean_ of the overall white matter and the global CMRG1c from these interrelated grey matter areas is also displayed in Fig. 1B (Pearson coefficient: − 0.577, *p* < 0.001). The Fazekas score was not correlated with the global CMRG1c from these interrelated grey matter areas (*p* = 0.13) although it was correlated with the ADC_mean_ of the overall white matter (Pearson coefficient: 0.481, p < 0.001).

**Table 2:** Volume and anatomical locations of the significant grey matter clusters obtained through SPM linear regression models aimed at correlating the CMRGlc of brain grey matter voxels with the ADC_mean_ values obtained from the overall white matter or from the white matter of each individual lobe, and with age and gender as covariables (*p*-value of 0.005, corrected for cluster volume (k = 39)).

	Volume (cm^3^)	Anatomical locations
Overall ADC_mean_	23.3	Internal temporal areas (BA 36), posterior associative junctions (BA 18-19-21-22-39-40), posterior cingulum (BA 30-31) and insulo-opercular areas (BA 11-13-47)
Frontal ADC_mean_	7.1	Left prefrontal dorso-lateral area (BA46), anterior cingulum (BA 32), right associative posterior junction (BA 39) and left insulo-opercular areas (BA 13, 41, 44, 45, 47)
Parietal ADC_mean_	23.0	Posterior associative junctions (BA 18-19-21-22-39-40), posterior cingulum (BA 30-31) and insulo-opercular areas (BA 13-45-47)
Temporal ADC_mean_	29.6	Posterior associative junction (BA 18-19-21-22-39-40), posterior cingulum (BA 23-24-30-31) and insulo-opercular areas (BA 13-44-45-47)
Occipital ADC_mean_	29.4	Internal temporal areas (BA 36), posterior associative junctions (BA 18-19-39-40), posterior cingulum (BA 30-31), and insulo-opercular areas (BA 13-44-45-47)

ADC_mean_, mean apparent diffusion coefficient; BA, Brodmann areas.

Further SPM analyses, obtained with the white matter of each individual lobe as opposed to the overall white matter, are provided in Table 2. Highly significant relationships were documented for the white matter of occipital and temporal lobes and to a lesser extent, of the parietal lobe, with the selection of grey matter sites being very close to those obtained with the ADC_mean_ of the overall white matter (see the description of these sites in Table 2 and corresponding SPM images in a supplementary file (Fig. 3)). By contrast, much poorer relationships were documented with the white matter of the frontal lobe.

### 3.3 Relationships with clinical and neuropsychological variables

As detailed in Table 3, the ADC_mean_ values of the overall white matter were significantly correlated with older age (*p* < 0.01), with a deterioration of both Gröber and Buschke (*p* < 0.01) and Trail Making tests (*p* < 0.02), as well as with higher peripheral systolic BP (*p* = 0.04) and higher central BP parameters (for systolic, p < 0.01 and pulse values, *p* = 0.02). Otherwise, no significant correlation was noted with Fazekas score (*p* > 0.14).

**Table 3:** Pearson coefficients between the ADC_mean_ of the overall white matter or the CMRGlc of areas correlated with global ADC_mean_, and clinical and BP variables and neuropsychological test scores.

	Global ADC_mean_	*p*-Value	CMRGlc	*p*-Value
Clinical and BP variables				
Age (years)	0.522⁎	< 0.01	− 0.315⁎	0.01
Duration of hypertension (years)	0.243	0.06	− 0.109	0.41
Number of antihypertensive medications	0.041	0.76	− 0.144	0.27
Peripheral systolic BP (mm Hg)	0.272⁎	0.04	− 0.210	0.11
Peripheral diastolic BP (mm Hg)	0.234	0.07	− 0.142	0.28
Peripheral pulse pressure (mm Hg)	0.194	0.14	− 0.178	0.17
Central BP (mm Hg)†	0.388⁎	< 0.01	− 0.362⁎	0.01
Central pulse pressure (mm Hg)†	0.302⁎	0.02	− 0.244	0.07
Neuropsychological test scores				
MMSE (/30)	− 0.173	0.19	0.262⁎	0.04
Benton score	− 0.045	0.73	0.056	0.67
Gröber and Buschke free recall (/48)	− 0.432⁎	< 0.01	0.498⁎	< 0.01
Gröber and Buschke cued recall	0.429⁎	< 0.01	− 0.406⁎	< 0.01
Trail making test A (sec)	0.435⁎	< 0.01	− 0.333⁎	< 0.01
Trail making test B (sec)	0.384⁎	< 0.01	− 0.518⁎	< 0.01
Trail making test (B-A) (sec)	0.300⁎	0.02	− 0.534⁎	< 0.01
Verbal fluency test score (P letter)	− 0.250	0.05	0.217	0.10
Verbal Fluency Test score (R letter)	− 0.235	0.07	0.202	0.12

BP, blood pressure; MMSE, mini-mental-state-examination; ADC_mean_, mean apparent diffusion coefficient; CMRGlc, cerebral metabolic rate of glucose.

⁎ For significant relationships with *p* < 0.05.

† Data available for only 56 subjects (24 women).

These relationships remained significant when ADC_mean_ values were replaced by the CMRGlc of the interrelated areas (Table 3), except that the relationship with the MMSE test became significant (*p* = 0.04) and that the central systolic BP became the sole significant BP parameter (*p* = 0.01).

**Fig. 2:**
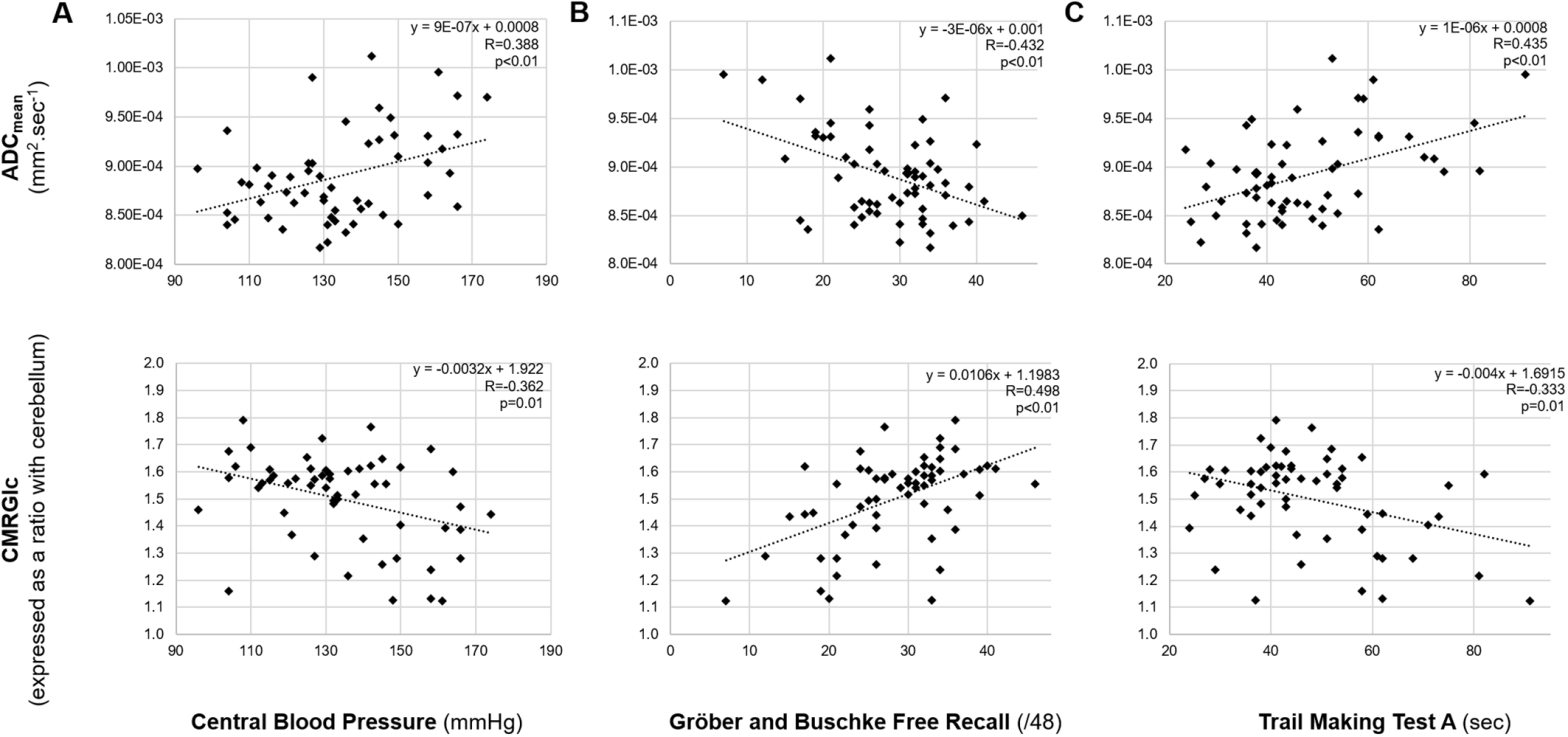
Linear relationships between the mean apparent diffusion coefficient (ADC_mean_) of the overall white matter (upper panel) or the cerebral metabolic rate of glucose (CMRGlc) of areas interrelated with overall white matter ADC_mean_ (lower panel) and the level of central systolic blood pressure (A), the Gröber and Buschke free recall test score (B) and the Trail Making Test A score (C).

Correlations between global ADC_mean_, CMRGlc of interrelated areas and the Gröber and Buschke free recall test, the Trail Making test and central blood pressure are illustrated in Fig. 2.

Detailed correlations between: 1) ADC_mean_ and CMRGlc from the different lobes and 2) clinical and neuropsychological scores and frontal, parietal, temporal and occipital white matter are provided in a supplementary file (Table 4).

## 4 Discussion

This dual DTI/^18^F-FDG-PET study shows that cross-sectional variations in the structure of the overall white matter are linked to the metabolism of Alzheimer-like cortical areas in a high-risk population of older hypertensive patients with only subjective memory complaints and thus, before the stage of any objective cognitive impairment. The clinical significance of these interrelated variations, as well as a possible contributing pathogenic role of hypertension, is strengthened by further observed relationships with neuropsychological tests and with central BP.

Multimodal DTI and FDG-PET imaging have already been reported in MCI or Alzheimer dementia patients (Sohn et al., 2015, Yakushev et al., 2011a, Yakushev et al., 2011b). The place of these two imaging modalities in the assessment of neurodegenerative disorders remain nevertheless debated, with the most efficient imaging method being DTI for certain authors (Zimny et al., 2015) on the one hand, and ^18^F-FDG-PET for others due to a closer link with cognitive impairment (Sohn et al., 2015). However, these two imaging methods do not provide the exact same information and, furthermore, our study shows that this interrelationship is already present at a very early stage of the development of cognitive impairment. Thus, it is likely that a better understanding of this interrelationship could constitute a key point for enhancing our knowledge on the development of neurodegenerative diseases.

The interrelationship between grey and white matter injuries are generally explained by the fact that any neuronal loss within the cortical areas commonly leads to a degeneration of the corresponding fiber tracts within the white matter (Bozzali et al., 2002). However, this cause-effect relationship can also likely occur in the other direction, notably with white matter vascular lesions documented at MRI which have been suggested to lead to a significant decrease in the metabolism of the frontal and temporal grey matter (Verger et al., 2016).

The determination of ADC_mean_ allows an assessment of the overall white matter volume without the need of a prior hypothesis on specific diseased sites, contrary to the determination of fractional anisotropy variations, which is mostly based on a region-of-interest approach, especially for the posterior cingulate and hippocampus (Bozoki et al., 2012, Fellgiebel et al., 2008, Walhovd et al., 2009, Zhuang et al., 2012). Using ADC_mean_, stronger influences on the CMRGlc of grey matter were documented herein for the white mater variations neighboring the grey-matter damages, i.e. occurring within the temporal and occipital lobes and to a lesser extent, within the parietal lobes (Table 2 and supplemental Fig. 3). Interestingly, the white matter lesions component of the global ADC_mean_ was not related to the CMRGlc of these interrelated grey matter areas nor with neurocognitive tests or blood pressure parameters. This likely suggests a predominant impact of white matter microstructural changes (which likely influence ADC values) over macrostructural vascular lesions (as assessed by the Fazekas score) on the interrelationships with the metabolism of grey matter Alzheimer areas.

A striking observation was that these interrelated grey matter areas mostly corresponded to typical Alzheimer's dementia hypometabolism patterns (Chen et al., 2011), in particular the internal temporal areas, the posterior associative junctions and the cingulum posterior (Brown et al., 2014). This finding would suggest that these interrelated white and grey matter variations could potentially constitute a very early stage of a cognitive neurodegenerative process, this consideration being furthermore strengthened by the clearly observed relationships with the results of the neuropsychological tests.

Indeed, the ADC_mean_ of the white matter, as well as the CMRGlc of the interrelated grey matter areas, were significantly correlated with the results of the Gröber and Buschke tests, yielding evidence of a link with memory functions. It should be pointed out that such correlations have previously been documented between these tests and equivalent imaging parameters, but only in populations involving patients with abnormal tests and suffering from Alzheimer's disease or MCI (Sali et al., 2013, Zimny et al., 2015). In the present study, these correlations were also observed for variations in the results of the Gröber and Buschke tests lying within the normal range, thereby constituting a highly original finding. The interrelated white and grey matter variations were additionally linked to a decrease in executive function, as assessed by the Trail Making tests. This latter observation is however not surprising since such functional decrease has already been documented in patients with low CMRGlc within temporal and parietal areas, as well as in those with white matter lesions, and presumably linked to an impaired connectivity with the frontal grey matter areas (Ewers et al., 2014, Scott et al., 2017).

Lastly, while the exact mechanism of these interrelated white and grey matters variations remains to be established, the observed relationships with BP level nevertheless strengthen the hypothesis of a contributing pathogenic role of hypertension. The central systolic BP level was indeed found herein to be a strong correlate for both the white matter ADC_mean_ and the CMRGlc of the interrelated grey matter areas. This finding is in keeping with the previous observations that central BP is a strong predictor of brain remodeling in the elderly as well as a sensitive indicator of cognitive performance not predicted by brachial pressures (Verger et al., 2015). In this setting, central BP has the advantage of being a more accurate reflection of the BP level found in cerebral arteries, comparatively to brachial BP which is dependent on a highly variable amplification of systolic BP from central to brachial arteries.

It remains to be determined whether this strong association with central BP is also documented in other populations of older subjects and especially those with no history of hypertension. Although a strong association with hypertension is well documented for grey matter hypometabolism as well as for future development of vascular dementia, it must be recognized that this association is less well established for neurodegenerative diseases. However, based on epidemiological, clinical (Gabin et al., 2017) and neuroimaging (Johnson and Albert, 2000) studies, certain authors have supported the hypothesis that Alzheimer's disease could primarily have a vascular-related mechanism. Furthermore, a longitudinal study found an association between antihypertensive treatments and a decrease in the rate of cognitive decline in two populations of hypertensive patients, one of which was treated with antihypertensive therapy (Tzourio et al., 1999). The present observations lend further support to the role of hypertension on not only the occurrence of white matter lesions but also on the decrease in the metabolism of certain grey matter areas, namely those evolving in parallel with white matter variations and occurring in regions corresponding to the current pattern of Alzheimer's disease. From a more practical viewpoint, these data suggest that hypertensive subjects with normalized central systolic BP may be at lower risk of further deteriorations not only of white matter but also of the grey matter areas involved in cognitive diseases, hence further supporting the interest of drugs aimed at lowering central pulse pressure.

The principal limitation of our study is its cross-sectional nature without any longitudinal follow-up. Thus, the impact of our imaging findings on the conversion into MCI or AD is presently unknown. Further longitudinal clinical trials conducted in populations at risk of cognitive decline and with sufficiently long follow-up periods are hence warranted. In addition, further comparisons with elderly patients, who are free of any hypertension and/or memory complaints, could be useful to accurately establish the interrelationships between hypertension, memory and results from PET/MRI imaging.

In conclusion, this dual DTI and ^18^F-FDG-PET study shows that cross-sectional variations in overall white matter structure are linked to the metabolism of Alzheimer-like cortical areas in older hypertensive patients, before the stage of objective cognitive impairment. The clinical significance of these variations is strongly supported by the concurrent observation of relationships with the results of cognitive tests, while the presence of further relationships with central BP strengthens the hypothesis of a contributing pathogenic role of hypertension.

The following are the supplementary data related to this article.

**Fig. 3:**
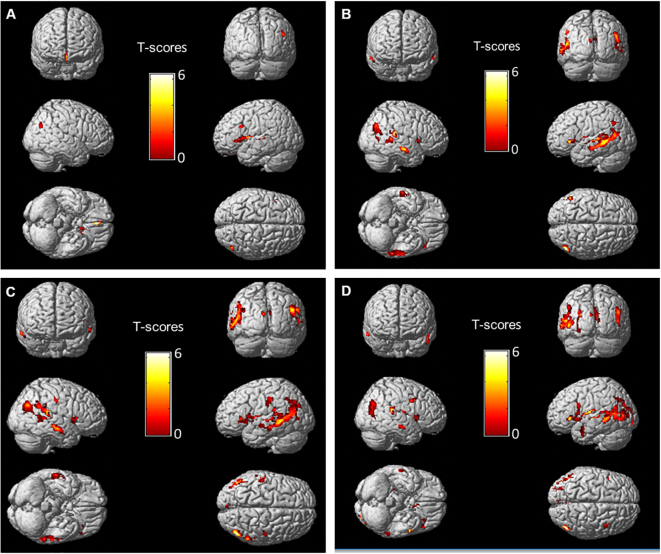
Three-dimensional volume rendering images representing the grey matter areas for which the metabolic rate of glucose (CMRGlc) was significantly (*p* < 0.005, with minimum cluster size of 39 voxels) and negatively correlated with the mean apparent diffusion coefficient (ADC_mean_) of the frontal (A), parietal (B), temporal (C) and occipital (D) white matter, using age and gender as covariates.

Table 4 Pearson coefficients for the correlations between 1) the ADC_mean_ and CMRGlc values from individual brain lobes and 2) clinical and BP variables as well as neuropsychological test scores. Table 4
