# Supplementary material for: Cross-sectional variations of white and grey matter in older hypertensive patients with subjective memory complaints
Source: Neuroimage Clin. 2017 Dec 18;17:804–10. doi: 10.1016/j.nicl.2017.12.024 (PMC5738235; doi:10.1016/j.nicl.2017.12.024)
Supplement: Table 4 — Pearson coefficients for the correlations between 1) the ADCmean and CMRGlc values from individual brain lobes and 2) clinical and BP variables as well as neuropsychological test scores. [file mmc1.docx]

**Supplementary Material (2)**

**Table 4: Pearson coefficients for the correlations between 1) the ADCmean and CMRGlc values from individual brain lobes and 2) clinical and BP variables as well as neuropsychological test scores**

|  | **ADC_mean_**  **Frontal** | **p value** | **ADC_mean_**  **Parietal** | **p value** | **ADC_mean_**  **Temporal** | **p value** | **ADC_mean_**  **Occipital** | **p value** | **CMRGlc**  **Frontal** | **p value** | **CMRGlc**  **Parietal** | **p value** | **CMRGlc**  **Temporal** | **p value** | **CMRGlc**  **Occipital** | **p value** |
| --- | --- | --- | --- | --- | --- | --- | --- | --- | --- | --- | --- | --- | --- | --- | --- | --- |
| **Clinical and BP variables** |  |  |  |  |  |  |  |  |  |  |  |  |  |  |  |  |
| Age (years) | 0.435* | *<0.01* | 0.488* | *<0.01* | 0.545* | *<0.01* | 0.443* | *<0.01* | -0.155 | *0.24* | -0.178 | *0.18* | -0.244 | *0.06* | -0.179 | *0.17* |
| Duration of hypertension (years) | 0.208 | *0.11* | 0.242 | *0.06* | 0.200 | *0.12* | 0.260* | *0.04* | -0.146 | *0.27* | 0.038 | *0.78* | -0.126 | *0.34* | -0.031 | *0.81* |
| Number of antihypertensive medications | 0.047 | *0.72* | 0.085 | *0.52* | 00028 | *0.83* | 0.070 | *0.60* | -0.202 | *0.12* | -0.118 | *0.37* | -0.149 | *0.26* | -0.036 | *0.78* |
| Peripheral Systolic BP (mmHg) | 0.167 | *0.20* | 0.232 | *0.08* | 0.203 | *0.12* | 0.152 | *0.25* | -0.132 | *0.32* | -0.109 | *0.41* | -0.245 | *0.06* | -0.301* | *0.02* |
| Peripheral Diastolic BP (mmHg) | 0.182 | *0.17* | 0.120 | *0.36* | 0.053 | *0.69* | -0.037 | *0.78* | -0.141 | *0.28* | -0.058 | *0.66* | -0.200 | *0.13* | -0.238 | *0.07* |
| Peripheral Pulse pressure (mmHg) | 0.090 | *0.49* | 0.224 | *0.09* | 0.235 | *0.07* | 0.235 | *0.07* | -0.073 | *0.58* | -0.104 | *0.43* | -0.183 | *0.16* | -0.231 | *0.08* |
| Central BP (mmHg) *^†^* | 0.304* | *0.02* | 0.429* | *<0.01* | 0.341* | *0.01* | 0.288* | *0.03* | 0.173 | *0.20* | -0.183 | *0.18* | -0.296* | *0.03* | -0.357* | *0.01* |
| Central Pulse pressure (mmHg) *^†^* | 0.294* | *0.03* | 0.353* | *0.01* | 0.297* | *0.03* | 0.282* | *0.04* | -0.050 | *0.71* | -0.108 | *0.43* | -0.152 | *0.26* | -0.186 | *0.17* |
| **Neuropsychological test scores** |  |  |  |  |  |  |  |  |  |  |  |  |  |  |  |  |
| MMSE (/30) | -0.226 | *0.08* | -0.172 | *0.19* | -0.150 | *0.25* | -0.143 | *0.28* | 0.090 | *0.50* | 0.114 | *0.39* | 0.206 | *0.12* | 0.154 | *0.24* |
| Benton score | -0.093 | *0.48* | 0.024 | *0.86* | 0.030 | *0.80* | 0.126 | *0.34* | -0.078 | *0.55* | -0.003 | *0.98* | 0.056 | *0.67* | -0.028 | *0.83* |
| Gröber and Buschke Free Recall (/48) | -0.456* | *<0.01* | -0.291* | *0.02* | -0.320* | *0.01* | -0.231 | *0.08* | 0.258* | *0.04* | 0.202 | *0.12* | 0.517* | *<0.01* | 0.247 | *0.06* |
| Gröber and Buschke Cued Recall | 0.447* | *<0.01* | 0.278* | *0.03* | 0.324* | *0.01* | 0.174 | *0.18* | -0.220 | *0.09* | -0.127 | *0.33* | -0.446* | *<0.01* | -0.193 | *0.14* |
| Trail Making Test A (sec) | 0.346* | *0.01* | 0.464* | *<0.01* | 0.370* | *<0.01* | 0.413* | *<0.01* | -0.048 | *0.72* | -0.062 | *0.64* | -0.311* | *0.02* | -0.259 | *<0.04* |
| Trail Making Test B (sec) | 0.287* | *0.03* | 0.392* | *<0.01* | 0.308* | *0.02* | 0.450* | *<0.01* | -0.235 | *0.08* | -0.360* | *0.01* | -0.525* | *<0.01* | -0.433* | *<0.01* |
| Trail Making Test B-A (sec) | 0.226 | *0.09* | 0.312* | *0.02* | 0.244 | *0.07* | 0.404 | *<0.01* | -0.274* | *0.04* | -0.426* | *<0.01* | -0.537* | *<0.01* | -0.441* | *<0.01* |
| Verbal Fluency Test score (P letter) | -0.252 | *0.05* | -0.200 | *0.13* | -0.050 | *0.70* | -0.176 | *0.18* | 0.217 | *0.10* | 0.041 | *0.76* | 0.327* | *0.01* | 0.124 | *0.35* |
| Verbal Fluency Test score (R letter) | -0.208 | *0.11* | -0.187 | *0.15* | -0.073 | *0.58* | -0.116 | *0.38* | 0.244 | *0.01* | 0.048 | *0.72* | 0.365* | *<0.01* | 0.135 | *0.31* |

*BP, Blood Pressure; MMSE, Mini-Mental-State-Examination; ADC_mean_, Mean Apparent Diffusion Coefficient; CMRGlc, Cerebral Metabolic Rate of Glucose*

**: for significant relationships with p<0.05. ^†^: data available for only 56 subjects (24 women)*
